# Cannabinoid receptors distribution in mouse cortical plasma membrane compartments

**DOI:** 10.1186/s13041-021-00801-x

**Published:** 2021-06-07

**Authors:** Hajar Miranzadeh Mahabadi, Haseeb Bhatti, Robert B. Laprairie, Changiz Taghibiglou

**Affiliations:** 1 Department of Anatomy, Physiology, Pharmacology; College of Medicine, University of Saskatchewan, 105 Wiggins Road, Health Sciences Bldg. Room GD30.5, Saskatoon, SK S7N 5E5 Canada; 2 College of Pharmacy and Nutrition, University of Saskatchewan, 105 Wiggins Road, Health Sciences Bldg. Room 3B36, Saskatoon, SK S7N 5E5 Canada; 3 Department of Pharmacology, College of Medicine, Dalhousie University, Halifax, NS Canada

**Keywords:** Type 1 cannabinoid receptor, Type 2 cannabinoid receptor, Lipid rafts

## Abstract

The type 1 and type 2 cannabinoid receptors (CB1 and CB2 receptors) are class A G protein-coupled receptors (GPCRs) that are activated by endogenous lipids called endocannabinoids to modulate neuronal excitability and synaptic transmission in neurons throughout the central nervous system (CNS), and inflammatory processes throughout the body. CB1 receptor is one of the most abundant GPCRs in the CNS and is involved in many physiological and pathophysiological processes, including mood, appetite, and nociception. CB2 receptor is primarily found on immunomodulatory cells of both the CNS and the peripheral immune system. In this study, we isolated lipid raft and non-lipid raft fractions of plasma membrane (PM) from mouse cortical tissue by using cold non-ionic detergent and sucrose gradient centrifugation to study the localization of CB1 receptor and CB2 receptor. Lipid raft and non-lipid raft fractions were confirmed by flotillin-1, caveolin-1 and transferrin receptor as their protein biomarkers. Both CB1 receptor and CB2 receptor were found in non-raft compartments that is inconsistent with previous findings in cultured cell lines. This study demonstrates compartmentalization of both CB1 receptor and CB2 receptor in cortical tissue and warrants further investigation of CB1 receptor and CB2 receptor compartmental distribution in various brain regions and cell types.

**Supplementary Information:**

The online version contains supplementary material available at 10.1186/s13041-021-00801-x.

## Introduction

Cannabinoid (CB) receptors are G protein-coupled receptors (GPCRs) that are highly expressed in almost all mammalian tissues. CB receptors are activated by the phytocannabinoid ∆^9^-tetrahydrocannabinol (Δ9-THC) and endogenous cannabinoids anandamide and 2-arachidonoylglycerol [1]. There are currently two widely-accepted CB receptors: the type 1 cannabinoid receptor (CB1 receptor) and the type 2 cannabinoid receptor (CB2 receptor). CB1 receptor is present in the central nervous system (CNS) and especially in the hippocampus, neocortex, basal ganglia and cerebellum [2, 3]. Interestingly, CB1 receptors are highly enriched in presynaptic and axonal compartments, with their more relevant activity in synaptic sites [4]. The CB1 receptor is involved in cognition, motor function, memory and nociception in the CNS [5]. The CB2 receptor is expressed at highest levels on immunomodulatory cells of the CNS—such as glia—and periphery—such as leukocytes [6, 7]. The expression of CB2 receptor is tightly regulated and induced by inflammatory signals in the microglia and brain-resident macrophages [8, 9].

The CB1 receptor is being intensively studied as a target of interest in a range of CNS disorders [10, 11] including anxiety [12], pain [13, 14], obesity [15, 16], multiple sclerosis [17, 18], nicotine addiction [19, 20], Parkinson disease [21, 22], Alzheimer disease [23] and Huntington disease [24]. The CB2 receptor has been associated with peripheral inflammatory disorders, including nephrotoxicity [25, 26]. Activation of the CB1 receptor at the cell surface typically results in the inhibition of cyclic adenosine monophosphate (cAMP) and the influx of Ca^2+^ via coupling with Gai/o proteins [27]. Intracellularly-localized CB1 receptors form a subpopulation with different functionalities from their cell surface-localized counterparts [28]. CB1 receptors in the endo/lysosome increase intracellular Ca^2+^ concentrations by releasing internal stores of Ca^2+^ and increase lysosomal permeability [29]. Mitochondrial CB1 receptors impair mitochondrial cellular respiration and production of cAMP, thus controlling cellular energy metabolism [28, 30]. Neuronal CB2 receptor has been mainly described as being localized within the postsynaptic region of the hippocampus [31]. Furthermore, previous studies have shown the intracellular localization of CB2 receptor in prefrontal cortical pyramidal neurons where it mediates neuronal excitability by controlling the Ca^2+^ activated Cl^−^ channels [32]. The CB2 receptor is also localized at the endo/lysosomes and modulates Ca^2+^ signaling [33].

Lipid rafts are dynamic microdomains in the cell membrane, composed of cholesterol and sphingolipids in comparison with the phospholipid-rich surrounding membranes. These compartments are involved in different cellular functions, including intracellular signaling, cellular polarity, membrane transport and molecule sorting [34–37]. Flotillins and caveolins are two structural proteins of lipid rafts. Acylated proteins and glycosylphosphatidylinositol (GPI)-anchor proteins are preferentially localized in lipid rafts. Cellular prion protein (PrPc) a GPI-anchor protein is specifically resided in lipid raft. Caveolin-1 is predominantly expressed in immature cortical neurons and has association with CB1 receptor [38]. Lipid raft proteins are relatively co-purified with sphingolipids, and cholesterol-rich membranes that are insoluble in cold non-ionic detergents followed by sucrose or Optiprep™ gradient fractionation [39–41]. To better understand the endocannabinoid system, the localization and distribution of CB receptors in the plasma membrane (PM) is important. The distribution of CB receptors in PM of rodent cortical tissue has not been adequately studied. In the present study, we thus investigated localization of CB1 and CB2 receptors in PM compartments of mouse cortices using cold Triton X-100 and sucrose gradient centrifugation method. Protein and lipid contents of PM contribute to distribution pattern of CB receptors in cell membrane. Since protein and lipid contents vary between cell lines and live tissue, we hypothesize that the distribution of CB receptors will be different from those previously reported cell lines studies. We found that the CB1 and CB2 receptors are localized in the non-lipid raft compartment of the PM of mouse cortical tissue.

## Materials and methods

### Chemicals and antibodies

Antibodies used in these experiments were as follows: rabbit polyclonal anti-CB1 receptor antibody directed against the first 99 amino acids of the receptor (Sigma-Aldrich, IgG, Cat# C1108, lot# SLCD8394) (dilution of 1:250) [42]; rabbit polyclonal anti-CB2 receptor antibody directed against the first 32 amino acids of the receptor (Abcam, IgG, Cat# ab3561, lot# GR45436-1) (dilution of 1:50) [43]; rabbit polyclonal anti-transferrin receptor (Abcam, IgG, Cat# ab84036) (dilution of 1:1000) [44]; rabbit polyclonal anti-flotillin-1 directed against residue surrounding Ile368 of human flotillin-1 (Cell Signaling, IgG, Cat# 3253) (dilution of 1:1000) [45]; mouse monoclonal anti-cytochrome C raised against amino acids 1–104 (Santa Cruz Biotechnology, IgG, Cat# sc-13156, lot# I1516) (dilution of 1:500) [46]; mouse monoclonal anti-stearoyl-CoA desaturase-1 (SCD1) (Abcam, IgG, Cat# ab19862, lot# GR138898-1) (dilution of 1:1000) [47]; rabbit polyclonal anti-caveolin-1 (Abcam, IgG, Cat# ab2910, lot# GR3382824-4) (dilution of 1:1000); mouse monoclonal anti-PrPc raised against N-terminal domain of PrPc (Abcam, IgG, Cat# ab61409, lot#GR3368463-1) (dilution of 1:1000) [48]. Anti-CB1 receptor antibody has been previously validated in our laboratory for western blot using both serial dilution and blocking peptide approaches to confirm the antibody’s specificity in rodent cortical tissue [49]. Anti-CB2 receptor antibody has been previously validated in Huang et al. [50] and Zhang et al. [51] for mouse lung and brain, respectively.

### Animals

Juvenile male C57BL/6 (5–7 weeks old, n = 5) mice were used in the study. Animal care protocols and guidelines were approved by the University of Saskatchewan Animal Research Ethics Board, following the Canadian Council on Animal Care (protocol# 20130062). Mice were housed 3 per cage in standard polypropylene cages in a temperature-controlled (21°C) colony room on a 12/12-h light/dark cycle and provided with ad libitum access to food, water, and environmental enrichment. Animals were anesthetized using 5% isoflurane and cortical tissue was collected on ice.

### Cell culture

The human epithelial mammary gland (MDA-MB-231) cell line was purchased from the American Type Culture Collection (ATCC, Cat# ATCC® HTB-26™) and cultured in the Roswell Park Memorial Institute 1640 medium (RPMI 1640, Thermofisher, Cat# 11875093) supplemented with 10% fetal bovine serum (FBS, Sigma Aldrich, Cat# F2442) and placed at 37 °C in a humidified incubator containing 5% CO2. Cells were grown to confluence in 100-mm dishes, washed in phosphate buffer saline (PBS) followed by isolation of PM compartments.

### Isolation of lipid-rafts compartment by sucrose-gradient centrifugation

Detergent-resistant membrane (DRM) was isolated based on the standard protocol described previously [52–55]. Mouse cortical tissues or MDA-MB-231 cell line were lysed with cold tissue homogenization buffer [150 mM NaCl, 20 mM Na_2_HPO_4_, 2 mM NaH_2_PO_4_, 20% (v/v) glycerol, 2 mM sodium orthovanadate and protease inhibitors (Roche, Cat# 04 693 159 001, lot# 37536800), pH 7.4)] by 30 strokes in a Dounce homogenizer, followed by 20 passages through a 22-gauge needle. Then centrifuged for 11 min at 12,000×g in 4 °C to clear cellular debris and nuclear material. The supernatant was centrifuged at 124,000×g (SW55 rotor) for 90 min at 4 °C to pellet the total PM. The pellet was resuspended in 2 mL cold solubilizing buffer containing 0.5% v/v Triton X-100 in Mes-Buffered Saline (MBS, 25 mM MES, pH 6.5, 150 mM NaCl), protease inhibitors and 2 mM sodium orthovanadate and incubated for 30 min at 4 °C. Incubation time is important for this step to decrease contamination from other subcellular organelles, including, endoplasmic reticulum and mitochondria [56]. Two mL of solubilized PM was mixed with 2 mL of 80% (w/v) sucrose and applied to the bottom of a 12 mL ultracentrifuge tube. The 30% sucrose was layered on top, followed by 4 mL of MBS buffer containing 5% sucrose. The sucrose gradient was centrifuged at 164,000 xg (SW41Ti rotor) for 16 h at 4 °C to isolate the lipid raft and non-raft compartments. Twelve equal fractions (1 mL each) were collected from the top of gradient to the bottom. A creamy white layer at the 5–30% interface was identified and collected as lipid raft fraction.

### Western blotting

Samples consisting an equal volume of each fraction were separated on 12% SDS-PAGE with the current in 90 V for 90 min and transferred onto polyvinylidene difluoride (PVDF) membranes (current 30 V, overnight). Membranes were incubated with 5% fat-free milk for 1 h at room temperature to block nonspecific background. The target proteins were immunoblotted with primary antibodies in 2% BSA overnight at 4 °C and then with corresponding HRP-conjugated secondary antibody for 1 h at room temperature. The membranes were exposed to enhanced chemiluminescence reagent (Bio-Rad) and imaged using ChemiDoc™ MP Imaging System. Western blot images were analyzed using NIH ImageJ software.

## Result

**Fig. 1:**
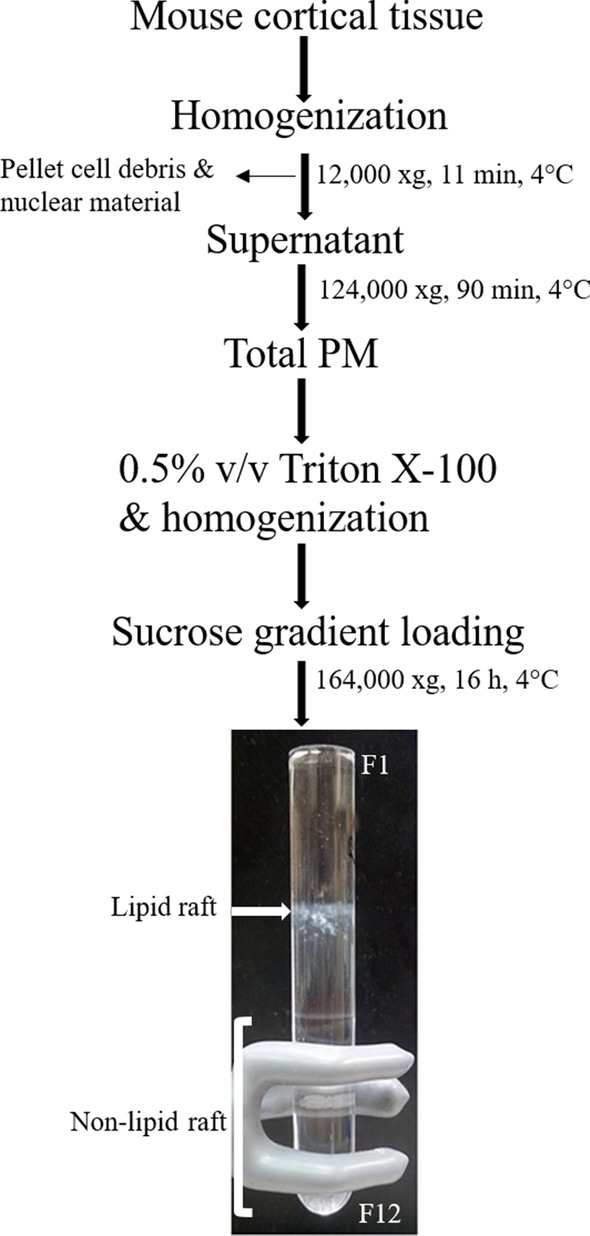
Isolation and identification of lipid rafts from non-raft fractions by discontinuous sucrose gradient centrifugation. Tissue samples were from juvenile male C57BL/6 mouse cortex. The lipid raft compartments are fully visible as an opaque band at the interface between 5 and 30% sucrose

**Fig. 2:**
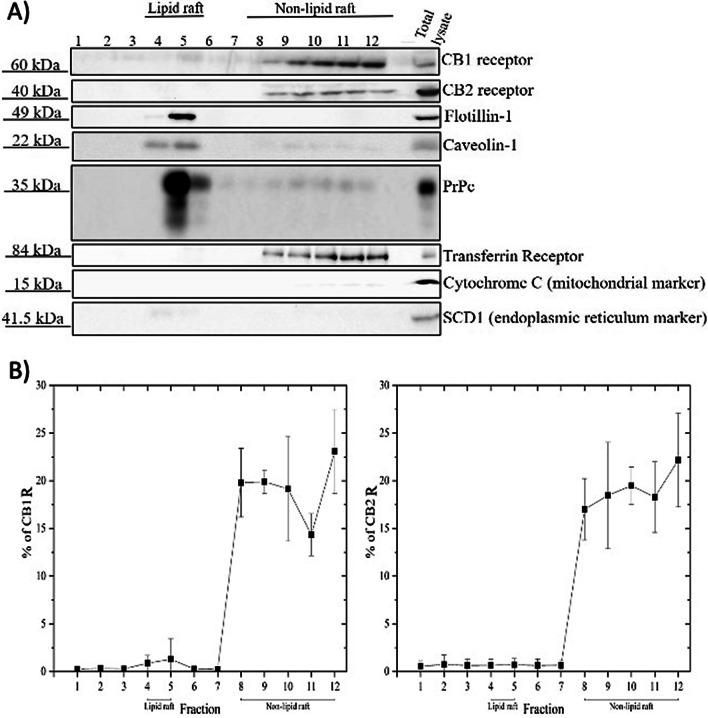
CB receptors PM compartmental distribution in mouse cortical tissue. **A** Aliquots of fractions collected from top to bottom of the gradient were subjected to SDS-PAGE isolation and analyzed by western blotting with antibodies directed against CB1 receptor, CB2 receptor, flotillin-1, caveolin-1, PrPc and transferrin receptor. To investigate purity of the fractionation process, the PVDF membranes were later stripped and probed with Cytochrome C (mitochondrial marker) and SCD1 (endoplasmic reticulum marker). Mouse cortical lysate (40 µg) was loaded on each gel as positive control. **B** Quantification of percent of CB1 or CB2 receptor present in each fraction relative to total receptor across all fractions within sample was performed using ImageJ software. All these experiments were repeated 5 times. Data are presented as mean ± S.E.M

We evaluated CB1 and CB2 receptors distribution and abundance through sucrose density gradient centrifugation in the PM of mouse cortical tissue and MDA-MB-231 cell line. Resistance to non-ionic detergent extraction (Triton X-100) at 4 °C and association with detergent resistant membrane (DRM) is one of the main biochemical features of lipid rafts components [40]. After the isolation process, an intact lipid raft fraction floating on the top of the centrifuge tube, fractions 4 and 5, was visible in comparison to non-raft fractions, 8–12, at the bottom of the tube (Fig. 1). Western blot analysis on an equal volume aliquot of each fraction indicated that the distribution of CB1 receptor in the PM is associated with non-raft fractions (fractions 8–12) (Fig. 2A, B). Similarly, CB2 receptor was present in high-density membrane fractions (fractions 8–12) (Fig. 2A, B). Previous data demonstrate that western blot detection of GPCRs, including the CB receptors, results in the detection of multiple bands corresponding to isoform variants, potential dimers, and post-translations modifications [49, 51, 57]. In our study, only the bands for CB1 receptor (60 kDa) and CB2 receptor (40 kDa) were quantified. Experiments were performed with 5 independent samples to determine the mean ± S.E.M % of CB1 or CB2 receptor present within each obtained fraction (Fig. 2B). Immunoblot analysis of individual fractions indicated that the lipid raft marker, flotillin-1, caveolin-1 and PrPc was present in fraction 4 and 5, confirming the isolation of DRM. The non-raft marker, transferrin receptor, was localized in soluble fractions 8–12. We also verified that PM fractionation was relatively purified without endoplasmic reticulum and mitochondria contamination by blotting with SCD1 and Cytochrome C, as their protein markers, respectively (Fig. 2A).

Detergent-resistant microdomains were also isolated from MDA-MB-231 cells. We found that CB1 receptor in MDA-MB-231 cells was Triton X-100 insoluble and the majority of the receptor floated in the fractions 4 and 5 of the gradients. In contrast, CB2 receptor appeared almost completely restricted at detergent-soluble fractions 8–12. Flotillin-1 and caveolin-1 in fractions 4 and 5 demonstrated the lipid rafts compartment and transferrin receptors in fractions 8 to 12, represented non-raft (bulk membrane) compartments (Additional file 1: Figure S1).

## Discussion

In the present study, we investigated the PM compartmental localization of CB1 and CB2 receptors in C57BL mouse cortical tissue and MDA-MB-231 cell line. In cortical tissue, both CB1 and CB2 receptors detected residing at the non-raft (bulk membrane compartments of PM). By contract, CB1 receptor enriched in lipid rafts of MDA-MB-231 cells, and CB2 receptor detected in non-lipid raft fractions.

The location of CB receptors in the PM helps to better understand the CB signalling pathway. The CB1 receptor cycles between the endosome and PM [58]. The role of lipid rafts/caveolae in compartmentalization of the endocannabinoid signalling machinery have been studied in several cultured cell systems [10]. These studies reported an association between the CB1 receptor with lipid raft/caveolae. In human embryonic kidney (HEK) 293 cells transfected with CB1 receptors, CB1 receptor internalization occurred, in parallel, through clathrin-coated pits and caveolae [59, 60]. CB1 receptor acylation at the C-terminal domain is necessary for proper interactions with lipid raft-associated G proteins [61–63]. In rat C6 glioma cells, the CB1 receptor co-localized with caveolin-1 [38]. Lipid raft disruption in rat C6 glioma cells by methyl-β-cyclodextrin (MCD) doubles CB1 receptor -dependent signaling through adenylyl cyclase and mitogen-activated protein kinases [64–66]. Moreover, in human breast cancer MDA-MB-231 cell line, the CB1 receptor associates with lipid raft/non-lipid raft fractions which relies on receptor activation/antagonism [67, 68]. CB1 receptor palmitoylation regulates membrane targeting and downstream signalling [69, 70]. Lipid pathway for ligand binding is essential for a CB receptor. Cholesterol acts as a CB1 receptor allosteric modulator [41]. These results point out that CB1 receptors are probably localized within lipid rafts [64, 66]. In contrast, in caveolin-1-lacking microglial cell line BV-2, only small amounts of CB1 receptor is found in lipid raft fractions [71]. Similar to our finding, the CB2 receptor has been shown to be localized in the non-lipid raft fraction of the dorsal root ganglion X neuroblastoma cell line (F-11) [72]. Proteins might translocate from non-raft to raft compartments through lateral movement along the PM. Different studies have shown that raft and non-raft segregation is a way to control protein function or determine protein destination. For example, the raft-localized neuronal Src is more active catalytically than in the soluble fraction [73]. Similarly, CB1 receptor -dependent signaling has been shown to be cell compartment specific in lipid rafts of HEK293 cells [60] and mitochondria [61]. In order to study this lateral movement of proteins, fluorescent molecular probes, which make lipid raft nanostructures visible through optical techniques can be helpful to find more details about the function of CB receptor in the PM. Further studies on CB receptors in PM are warranted to discover lateral movement for these receptors.

Our result from MDA-MB-231 cell line support previous studies related to the CB1 and CB2 receptors distribution in lipid raft and non-raft compartment respectively [67]. On the other hand, our findings indicate that CB1 and CB2 receptors are not co-localized with lipid rafts in mouse cortical tissue, which is in disagreement with the previous findings in cell lines. This inconsistency may arise from the difference between actual brain tissue used in our study with the mono-layer cell lines. Since mature neurones express less caveoline-1, it might decrease CB1 receptor association with lipid raft compartments of the cortical cell membrane. It has yet to be determined if caveolin-1 low expression affects the interaction of CB1 receptor with cortical tissue lipid rafts. This connection could explain why CB1 receptors are found in different PM compartments in different cell types. Moreover, The CB2 receptors were recovered in the higher density portion (non-rafts) of the gradient. It has been previously reported that membranes from different cells display altered average densities [40]. To avoid this average density alteration, we took advantage of using freshly dissected cortices. Our data describe the localization of CB receptors in juvenile, male, drug-naïve cortical tissue from mice and form a baseline for future research. These studies warrant more further investigations on PM compartmental distributions of CB1 and CB2 receptors in various brain anatomical sections.

## Supplementary Information

**Additional file 1: Figure S1.** CB receptors PM compartmental distribution in MDA-MB-231 cell line. Aliquots of fractions collected from top to bottom of the gradient were subjected to SDS-PAGE isolation and analyzed by western blotting with antibodies directed against CB1 receptor, CB2 receptor, flotillin-1, caveolin-1 and transferrin receptor. MDA-MB-231 cell lysate (40 µg) was loaded on each gel as positive control.

## Data Availability

Please contact author for data requests.
