# Supplementary material for: Cannabinoid receptors distribution in mouse cortical plasma membrane compartments
Source: Mol Brain. 2021 Jun 7;14:89. doi: 10.1186/s13041-021-00801-x (PMC8183067; doi:10.1186/s13041-021-00801-x)
Supplement: Supplementary file 1 — Additional file 1: Figure S1. CB receptors PM compartmental distribution in MDA-MB-231 cell line. Aliquots of fractions collected from top to bottom of the gradient were subjected to SDS-PAGE isolation and analyzed by western blotting with antibodies directed against CB1 receptor, CB2 receptor, flotillin-1, caveolin-1 and transferrin receptor. MDA-MB-231 cell lysate (40 µg) was loaded on each gel as positive control. [file 13041_2021_801_MOESM1_ESM.docx]

Additional Material:


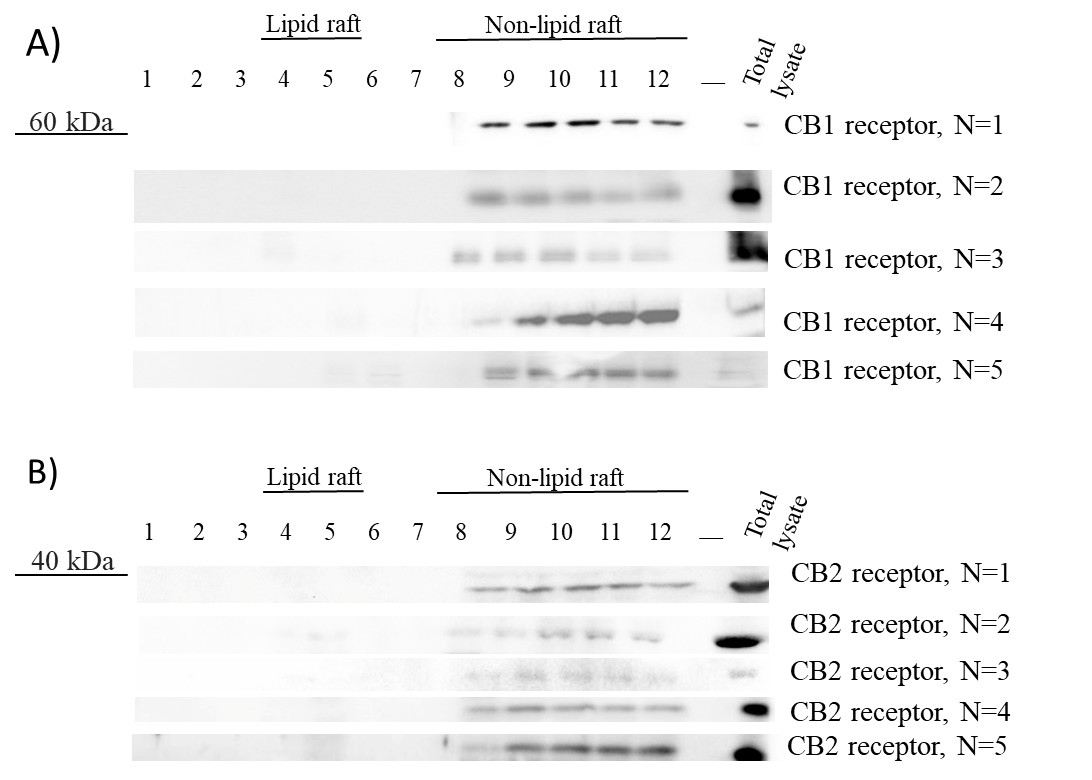


**Additional file 1: Figure S1. Raw data for CBRs. CB receptors distribution in the lipid raft and non-raft fractions.** A, B) we repeated lipid raft isolation (N=5) to investigate the CB receptors distribution in the plasma membrane of cortical tissues.
